# Public beliefs about trauma and its consequences: Profiles and correlates of stigma

**DOI:** 10.3389/fpsyg.2022.992574

**Published:** 2023-01-04

**Authors:** Joshua D. Clapp, Alexandria F. Sowers, Scott A. Freng, Layla M. Elmi, Robert A. Kaya, Alicia R. Bachtel

**Affiliations:** Department of Psychology, University of Wyoming, Laramie, WY, United States

**Keywords:** trauma, stereotype (psychology), stigma, attitudes, beliefs and assumptions, social processes

## Abstract

Public stereotypes about trauma exposure and its likely consequences have the potential to influence levels of support extended to survivors in the larger community. The current project sought to examine unique profiles of stereotype endorsement both within and across participants sampled from distinct populations. Trauma-related stereotypes involving symptom course, dangerousness, employability, social functioning, predictability, character, and treatment need were examined in undergraduate (*N*_1_ = 404; *N*_2_ = 502) and MTurk (*N*_3_ = 364) samples. Sympathizing [low overall endorsement], Fearful [high overall endorsement], Pejorative [high endorsement + moralizing beliefs], Safety-Focused [intermediate endorsement + dangerousness], and Performance-Focused [intermediate endorsement + employability] groups were replicated in latent profile models across all samples. Stereotype profiles demonstrated hypothesized associations with general perspectives of mental illness although support for consistent relations with respondent characteristics (e.g., sex; personal exposure to trauma; reported exposure in friends/family) was limited. Data suggest that trauma stereotypes are endorsed at high frequencies in the general community and conform to systematic patterns of prejudice that may be overlooked in more global assessments of stigma.

## 1. Introduction

Decades of research on public attitudes toward individuals with psychological difficulties has contributed to the recognition of mental health stigma as a topic of broad relevance to clinical science (for reviews see Corrigan, 2004; Hinshaw and Stier, 2008; Abdullah and Brown, 2011; Parcesepe and Cabassa, 2013). Whereas studies confirm the continuing endorsement of stereotyped beliefs and prejudicial behavior in members of the general community (Martin et al., 2000; Schomerus et al., 2012; Kvaale et al., 2013), existing data are largely limited to perspectives on a handful of conditions (e.g., schizophrenia, depression, alcohol and substance abuse), leading to questions about possible problem-specific profiles of stigmatization. Trauma exposure is not a mental health condition, but it *is* an experience associated with risk for behavioral difficulties and negative societal perceptions (e.g., Holguin and Hansen, 2003; Nash et al., 2009; Dworkin et al., 2019). Empirical research and models of trauma-related difficulties identify support from family, friends, and the larger community as a critical factor for resilience (e.g., Brewin et al., 2000; Maercker and Horn, 2013), making public beliefs about trauma and its potential consequences relevant to the study of recovery. However, research in this area is generally restricted to the measurement of perceived stigma as reported by survivors, limiting a clear picture of what members of the community believe, how often specific beliefs are endorsed, and the stability of trauma stereotypes across subsets of the larger population. The aims of the current project were to (a) determine the extent to which members of the public endorse negative beliefs about trauma and its consequences, (b) identify conceptually and clinically relevant patterns of stereotype endorsement, and (c) examine the associations of belief profiles with person-level characteristics and global attitudes toward mental illness.

Mental health stigma refers to a collection of attitudes and beliefs that motivate members of the public to fear, reject, avoid, and discriminate against individuals who have – or are believed to have – emotional and behavioral difficulties (Parcesepe and Cabassa, 2013). An influential framework developed by Corrigan (2000, 2004, 2007) organizes stigmatization into processes related to the cuing, stereotyping, prejudice, and discrimination of those with mental health concerns. In this model, cues refer to a signal or mark that identify an individual as someone struggling with mental illness. Cues include both observable characteristics (e.g., behavioral symptoms, skill deficits, unusual appearance) as well as covert signals derived from socially-constructed labels (e.g., psychiatric diagnoses, associations with mental health services). Cues are believed to activate stereotypes, defined as knowledge structures acquired through shared socialization. Common stereotypes of individuals with mental health difficulties include presumptions of dangerousness, incompetence, unpredictability, moral weakness, and the need for monitoring and/or social restriction (e.g., Martin et al., 2000; Corrigan et al., 2005; Feldman and Crandall, 2007; Hinshaw and Stier, 2008). The acceptance of mental health stereotypes results in prejudice which is thought to trigger negative emotion (e.g., fear, anger, disgust) in the stigmatizer. Finally, prejudicial attitudes are proposed to elicit discriminatory behavior toward the stigmatized outgroup. It is important to note that discrimination against those suspected of having mental health difficulties can include both overt, intentional behavior (e.g., social exclusion) as well as more covert and potentially unintended actions (e.g., lowered expectations for performance in educational and occupational settings; Holguin and Hansen, 2003; Link et al., 2004; Angermeyer and Schomerus, 2017).

An extensive literature documents the impact of public stigma on the employment, housing opportunity, symptom severity, healthcare access, social functioning, and quality of life of individuals with mental health concerns (e.g., Corrigan, 2007; Corrigan and Shapiro, 2010; Kvaale et al., 2013). Indeed, a comprehensive review by Hinshaw and Stier (2008) concludes that the functional consequences of mental health stigma may surpass the direct impact of psychological conditions themselves. However, a critical examination of this literature identifies areas for continued growth. One limitation involves a narrow emphasis on public perceptions of schizophrenia, depression, and alcohol/substance use disorders (e.g., Angermeyer and Dietrich, 2006; Angermeyer and Schomerus, 2017). Investigators have noted that (a) specific stereotypes and the severity of public discrimination against those with mental health difficulties differ from condition-to-condition, and (b) the endorsement of domain-specific beliefs (e.g., perceptions of dangerousness versus incompetence versus moral failing) is likely to vary both within and across individuals (Corrigan, 2004; Hinshaw and Stier, 2008; Parcesepe and Cabassa, 2013). As a result, research on stereotypes involving specific, stigmatized populations is needed to understand beliefs held by the public and the implications these perceptions may have on the targets of stigma.

Exposure to significant trauma involving actual or threatened death, serious injury, and/or sexual violence presents an interesting application of Corrigan’s social-cognitive model. To be clear, trauma exposure is not a mental illness. It is an experience that is common in the general population (estimates for lifetime prevalence of trauma exposure range from 51.2 to 70.4%), and – while impactful – is an event that most will respond to with trajectories of natural recovery (estimates for lifetime prevalence of posttraumatic stress range from 7.8 to 12.9%; Kessler et al., 1995, 2017). However, much of the general public is likely to associate trauma exposure with chronic mental health concerns and/or irreparable harm (e.g., “They’ll never be the same”). Many traumatic events are themselves linked to broad social stigmatization (e.g., child abuse, sexual assault, combat exposure), multiplying opportunities for the application of stereotypes and rejection. Conceptual frameworks of post-trauma responding consistently identify support from family, friends, and the larger community as a central component of recovery (Charuvastra and Cloitre, 2008; Maercker and Horn, 2013). Existing models of mental health stigma may help to clarify how members of the general public – including those serving as potential support members – view trauma and its consequences, and how these beliefs may influence adaptation and recovery in survivors.

From the perspective of Corrigan’s (2004, 2007) social-cognitive model, multiple aspects of the trauma experience provide cues for potential stigmatization. Distress, withdrawal, and other reactions that are normative in the immediate aftermath of exposure could trigger presumptions of mental illness in members of a survivor’s community. It is also possible for the *absence* of these signals to be interpreted as signs of disruption. Results of the larger literature indicate that ambiguous and/or otherwise normative behavior is routinely interpreted as evidence of mental illness when viewed within the context of socially-constructed labels (Holguin and Hansen, 2003; Hinshaw and Stier, 2008). Because many forms of trauma exposure are publicly known within a survivor’s community (e.g., traumatic accidents, publicized assaults), demonstrations of true resiliency may be interpreted as the “suppression” of underlying psychological damage. Survivors are then caught in a catch-22 where both the presence and the absence of distress following exposure can be interpreted as evidence of pathology. It is also worth noting that individuals who have not been exposed – but who are *suspected* of experiencing trauma by members of the larger community (e.g., children from “bad homes,” service members returning from deployment) – can be caught in this cycle. The result is that assumptions of profound and irreversible psychological damage may be applied regardless of symptoms, behaviors, or actual trauma status.

Stereotypes of mental health difficulties involving perceptions of dangerousness, unpredictability, incompetence, moral deficits, and the need for intervention (e.g., Martin et al., 2000; Link et al., 2004) are likely to be activated by real or perceived cues of traumatization, with the endorsement of stereotyped beliefs resulting in prejudicial attitudes. Prejudice, in turn, is expected to motivate various forms of discriminatory behavior. In a review of trauma portrayals in the news media, Purtle et al. (2016) report that nearly half of employers (46%) in a 2010 survey by the Society for Human Resource Management identified posttraumatic stress disorder (PTSD) as a perceived barrier to the hiring of former military service members. Experimental studies evaluating discriminatory behavior toward survivors of childhood sexual abuse suggest that educators hold lower expectations of labeled children and view them as less likely to succeed relative to unlabeled students (Bromfield et al., 1988). Research with emerging adults offers further evidence of lowered expectations for hypothetical survivors of child sexual abuse as well as assumptions of psychological difficulties and persistent social dysfunction (Briggs et al., 1994, 1995). Results are consistent with discriminatory behaviors reported in other survivor populations (e.g., Ullman, 1999; Dworkin et al., 2019), suggesting that more targeted research on community beliefs about trauma and its potential consequences is needed.

Stigmatization has received considerable attention in the larger trauma literature with research addressing issues related to self-stigma (e.g., Deitz et al., 2015; Barr et al., 2019), stigmatization as a barrier to care (e.g., Brown and Bruce, 2016; Kantor et al., 2017), correlates of perceived stigma (e.g., Pietrzak et al., 2009; Soomro and Yanos, 2019), social reactions to the disclosure of trauma (e.g., Ullman, 2011; Dworkin et al., 2019), and anti-stigma interventions (e.g., Gould et al., 2007; Nickerson et al., 2020). What is less clear, however, are the specific beliefs that members of the general community – those likely to be friends, family members, coworkers, acquaintances, and romantic partners of survivors – hold about trauma and its potential consequences. Whether prejudicial beliefs *exist* in larger society is not in question. Research with survivors provides clear evidence for prejudice and discrimination from both support members and the community in general (e.g., Ullman, 1999; Tener and Murphy, 2015; Dworkin et al., 2019). However, existing data rely almost exclusively on survivor reports of experienced or perceived stigmatization which offers limited information on the potential scope of the issue. The assessment of survivor-reported stigmatization also tends to focus on overtly pejorative reactions that fail to capture more subtle – but still problematic – stereotyped beliefs (e.g., lowered expectations, presumed need for psychological intervention). As such, the aims of the current, multi-study project were (a) to examine the extent to which members of the general community endorse stereotyped beliefs about trauma and its consequences, (b) to identify conceptually relevant and generalizable patterns of belief endorsement, (c) to determine the degree to which belief profiles are linked to respondent exposure and/or affiliation with individuals who are trauma exposed, and (d) to assess whether profiles of endorsement correspond to more generalized perceptions of mental health difficulties.

## 2. Study 1

The aims of the initial study were to examine overall rates of belief endorsement and to identify unique profiles of person-specific responding across stereotype domains. Individuals in the larger community almost certainly vary both in *the degree* to which they hold stereotyped beliefs about trauma and in *the kinds of consequences* they believe trauma may have. The current project takes a person-centered approach to assessing profiles of endorsement that reflect unique patterns of assumptions/concerns. Similar to traditional cluster analyses, models in this study were used to identify subgroups of respondents characterized by similar profiles of belief endorsement (see section “2.1.3 Analytic plan”). Data were also used to examine the associations of stereotype profiles with respondent characteristics and the extent to which patterns of endorsement may be attributable to confounding factors such as impression management and state-level mood.

### 2.1. Methods

#### 2.1.1. Participants

Participants were university students (*N* = 404) completing studies for research credit in undergraduate psychology courses.^1^ All measures were administered online. No exclusion criteria were implemented given efforts to maximize variability in the endorsement of trauma-related stereotypes. Data collection procedures for this and subsequent studies were approved through the University of Wyoming Human Subjects Institutional Review Board. Participants in the initial study identified predominantly as female (72.0%) and White/Non-Hispanic (81.7%). Mean age of the sample was 19.5 years (SD = 2.4). Full background characteristics are provided in Table 1.

**TABLE 1:** Background characteristics.

	Study 1	Study 2	Study 3
*N*	404	502	364
Sex (% female)	72.0%	68.7%	65.1%
Age	19.5 (2.4)	19.9 (3.4)	35.9 (13.3)
White/Non-Hispanic	81.7%	77.3%	76.1%
**Geographic region**			
– West	100%	100%	19.5%
– Southwest	–	–	12.6%
– Midwest	–	–	20.6%
– Southeast	–	–	28.6%
– Northeast	–	–	17.3%
**Education**			
– 12th grade or less	–	–	8.2%
– Some college	100%	100%	32.4%
– 4-year degree	–	–	31.6%
– Graduate coursework	–	–	27.5%
– Current student	100%	100%	22.7%
**Employment**			
– Full-time	–	–	51.6%
– Part-time	–	–	20.3%
– Unemployed	–	–	15.1%
– Other	–	–	12.9%
Prior treatment (% yes)	22.8%	25.3%	49.0%
Friend/family exposure (% yes)	41.8%	41.8%	52.5%
Probable trauma history (% yes)^a^	25.0%	35.1%	38.5%
TBS total	35.0 (16.2)	33.5 (15.4)	36.1 (17.0)

TBS total, total score from the Trauma Beliefs Scale indicating percentage of items endorsed.

^a^History of probable trauma assessed with a single item in Study 3.

#### 2.1.2. Measures

##### 2.1.2.1. Trauma belief scale

The TBS is a descriptive, 51-item survey of stereotyped beliefs involving trauma exposure and its potential consequences. Items were developed from the authors’ previous work with survivors, support members, and the general public (e.g., Clapp and Beck, 2009; Clapp et al., 2014, 2022; Kern et al., 2019), with stereotypes mapping on to specific domains of mental health stigma described in the larger literature (Farina, 1981; Corrigan, 2004; Link et al., 2004; Hinshaw and Stier, 2008; Parcesepe and Cabassa, 2013). Items related to *Course* involve the degree to which the consequences of trauma are seen as persistent and irreversible (e.g., *People exposed to serious trauma are damaged*). *Dangerousness* refers to perceptions of survivors as inherently aggressive (e.g., *People exposed to serious trauma often become violent*). *Employability* relates to lowered expectations of survivors in work settings (e.g., *People exposed to serious trauma are unable to do their jobs as effectively as before*) whereas *Social Concerns* involve anticipated impairment in social domains (e.g., *It is difficult to be friends with someone who has experienced serious trauma*). *Moralizing* beliefs place blame on survivors for post-trauma reactions (e.g., *People who have difficulty moving past serious trauma are generally looking for attention*). *Predictability* relates to concerns with the reliability and emotional stability of survivors (e.g., *People exposed to serious trauma are generally untrustworthy*). Finally, *Mental Hygiene* refers to the belief that trauma exposure necessitates involvement in formal mental health treatment (e.g., *People exposed to serious trauma need to be on medication*). Responses to the TBS are collected using a dichotomous True/False format (1, 0) with items preceded by the following instructions:

*People may be exposed to stressful events during their lifetime. Some of these events can be classified as serious trauma (e.g., physical or sexual abuse, rape, exposure to death or injury during military service, involvement in accidents where someone was killed and/or seriously injured). Please read the following statements about the consequences of serious trauma. Rate each item as true or false based on your own beliefs or experiences. All responses are confidential, and there are no right or wrong answers. We are interested only in your personal opinion.*

A copy of the full survey with subsequent domain codes is available in Supplementary Appendix A.

##### 2.1.2.2. Exposure screening protocol

The ESP (Clapp et al., 2019) is a minimally invasive, self-report checklist developed to identify potential exposure to Criterion-A trauma. Respondents are instructed to indicate whether they have directly experienced any of five traumatic events that occur at some frequency in the general population (i.e., disaster, fire, traffic accidents, physical assault, sexual assault). A final, open-ended item allows for the endorsement of other forms of exposure not included in the set list of events. Respondents are asked to provide contextual information for each endorsed event including age(s) of occurrence; age of most severe exposure; whether the event resulted in injury to the individual or others; subjective emotion at the time of exposure; and ratings of ongoing distress as a consequence of the event (1 = *none*, 5 = *extremely*). Initial psychometric data provides strong support for the ESP as a screen-in measure for Criterion-A trauma, with 96.7% of individuals identified for inclusion in the original development study (i.e., those reporting probable trauma and ongoing distress at a 2 or higher) verified as experiencing one or more Criterion-A events in subsequent clinical interviews (Clapp et al., 2019). Based on this scoring, 25.0% of the current sample reported at least one experience consistent with potential Criterion-A trauma (disaster = 2.2%; fire = 0.7%; traffic accidents = 14.1%; physical assault = 4.0%; sexual violence = 7.4%; other trauma = 2.5%).

##### 2.1.2.3. Treatment history and family/friend exposure

Single-item ratings were administered to determine participant history of psychological treatment (*Have you ever taken medication or received counseling for a psychological issue?*) as well as trauma exposure in potential support members (*Do you have a close friend or family member who has experienced a significant trauma?*). Responses were collected using a dichotomous Yes/No (1, 0) format.

##### 2.1.2.4. Positive and negative affective schedule

The PANAS (Watson et al., 1988) is a 20-item measure developed to assess dimensions of positive and negative mood. Descriptors indicative of positive and negative emotionality are rated on a 1 (*Very slightly or not at all*) to 5 (*Extremely*) scale. State-level positive and negative affect is calculated as the mean of relevant items scaled by a multiplier of 10 (range = 10–50). Psychometric evaluation of the PANAS provides support for the convergent, discriminant, and factorial validity of scores (Watson et al., 1988). Internal consistency of positive (α = 0.91) and negative (α = 0.86) scales were excellent in these data.

##### 2.1.2.5. Marlowe–Crowne social desirability scale

The MCSDS is a 33-item self-report measure of socially desirable responding (Crowne and Marlowe, 1960). True-false items (1, 0) are summed to form a total score, with higher values interpreted as evidence of greater social desirability. MCSDS scores have shown evidence for adequate reliability and validity in undergraduate respondents (Loo and Thorpe, 2000). The internal consistency of scores in the current sample was acceptable (α = 0.76).

#### 2.1.3. Analytic plan

Patterns of endorsement across stereotype domains were examined in a series of latent profile analyses (LPA). LPA is a person-centered modeling technique allowing for the extraction of unobserved subgroups characterized by common profiles of responding (Goodman, 2002). Data for these analyses included domain scores from the TBS, calculated as the percentage of items endorsed for Course, Dangerousness, Employability, Social Concerns, Moralizing, Predictability, and Mental Hygiene domains (i.e., # items endorsed/# items*100). Resulting scores range from 0 to 100 with higher values reflecting greater endorsement of stereotyped beliefs. LPA were conducted in MPlus 8.2 (Muthén and Muthén, 1998/2018) using maximum likelihood estimation with robust standard errors (MLR). Results classify participants to the profile that is most consistent with their specific pattern of responding. Consistent with best-practice methods for LPA (Nylund et al., 2007), successive models containing an increasing number of profiles were considered for these data. Final model selection was guided by the interpretive value of solutions as well as statistical fit. Fit indices included the Akaike information criterion (AIC), Bayesian information criterion (BIC), Bootstrapped Likelihood Ratio Test (BLRT), and entropy criteria. AIC and BIC are standard information criteria where lower values represent incremental improvement in model fit. BLRT, by contrast, compares an estimated model with a solution containing *c*-1 classes. Low *p*-values suggest statistical gains relative to the more parsimonious model. Entropy provides an index of the degree to which profiles are uniquely characteristic of a given class. Values ≥ 0.80 are indicative of adequate profile separation (Lubke and Muthén, 2007).

The associations of final profile membership with person-level variables including sex, probable Criterion-A exposure, treatment history, and reported trauma in friends or family were examined to contextualize the characteristics of stereotype groups. The associations of profile membership with state mood and social desirability were also examined to assess potential confounds to interpretation (e.g., stereotype profiles attributable to socially desirable responding and/or state-level mood versus actual beliefs about trauma and its potential consequences). Effect sizes for omnibus tests are presented as Cramér’s *V* (small: *V* = 0.10; medium: *V* = 0.30; large: *V* = 0.50) and η^2^ (small: η^2^ = 0.01; medium: η^2^ = 0.06; large: η^2^ = 0.14) for categorical and continuous variables, respectively (Cohen, 1988). Coefficients for pairwise tests are given as Cohen’s *d* (small: *d* = 0.20; medium: *d* = 0.50; large: *d* = 0.80) with estimates standardized using the square root of the pooled variance of target groups. All analyses were conducted in Stata 15.1 (StataCorp, 2017).

Analyses also incorporated a number of recommendations by the American Statistical Association for enhanced reporting of scientific research (Wasserstein and Lazar, 2016; Wasserstein et al., 2019). Given that *p*-values can be interpreted as an index of the extent to which data are incompatible with an underlying null model, exact values for tests with estimates less than 0.001 are reported in scientific notation. Supplemental statistics including Shannon information values (*s*-values; Greenland, 2019) and Bayes Factor Bound (BFB; Benjamin and Berger, 2019) are also presented to facilitate inferences regarding the strength of evidence for observed effects. *s*-values are a non-linear transform of *p* (*s* = |ln(*p*)/ln(2)|) that provides roughly the same evidence *against* the null hypothesis as would observing *s* successive “heads” in flips of a hypothesized fair coin (e.g., evidence against H_0_ for *p* = 0.05 [*s* = 3] is similar to evidence *against* a fair coin that could be inferred following three successive “heads”). BFB is an alternative transform (BFB = 1/[−e*p*ln(*p*)]), representing the *largest possible* Bayes factor consistent with the data. Estimates can be interpreted as a best-case scenario of the odds for H_1_ relative to H_0_ (e.g., for *p* = 0.05 [BFB = 2.46], the odds of H_1_ *are at most* 2.5 times the odds of H_0_ given the data provided). Interpretive benchmarks for Bayes factors (1–3 = Not worth more than a bare mention; 3–20 = Positive; 20–150 = Strong; >150 = Very Strong; Kass and Raftery, 1995) may also be applied to estimates of BFB. Supplemental coefficients are viewed as a means of augmenting statistical interpretation and to provide a more balanced assessment of the strength of evidence for individual effects (Wasserstein et al., 2019).

### 2.2. Results

#### 2.2.1. Stereotype endorsement

**FIGURE 1:**
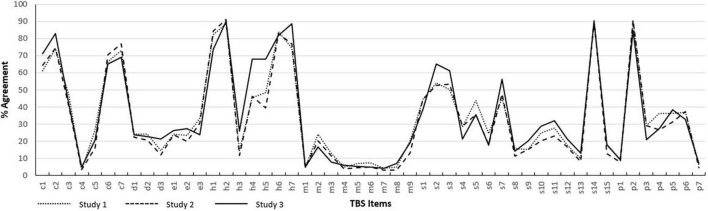
Item-level endorsement for Study 1, Study 2, and Study 3. Item-level data is presented in Supplementary Appendix A.

The inspection of item-level data indicated clear variability in the endorsement of sampled beliefs (see Figure 1). Agreement with stereotypes involving course, mental hygiene, and social concern domains was relatively common. The endorsement of moralizing, dangerousness, and employability stereotypes was lower although agreement with pejorative content remained notably high (e.g., *People who have difficulty moving past serious trauma are emotionally weak* [24.3%]; *People exposed to serious trauma often become violent* [24.0%]; *It is difficult to work with someone who has experienced serious trauma* [24.0%]). Item-level responses are summarized in Supplementary Table A.

#### 2.2.2. LPA

Results of the LPA suggested possible solutions containing four to six profiles. The 4-profile model (AIC = 24,712.7; BIC = 24,864.8; BLRT *p* < 0.001; entropy = 0.94)^2^ identified conceptually distinct subgroups that included participants with relatively low levels of stereotype endorsement; participants with high endorsement aside from moralizing; and a pair of intermediate profiles distinguished by unique elevations on dangerousness- versus employability-related beliefs. Subgroups in the 5-profile solution (AIC = 24,602.7; BIC = 24,786.8; BLRT *p* < 0.001; entropy = 0.94) were identical to those in the previous model along with the extraction of a final profile demonstrating uniformly high endorsement across all stereotype domains. Results of the final 6-profile model (AIC = 24,526.1; BIC = 24,742.1; BLRT *p* < 0.001; entropy = 0.88) built on those of the initial solutions, although the novel class extracted in this analysis mirrored the original low-endorsement group with only a nominal increase in overall elevation. As expected, AIC and BIC values decreased across successive models. BLRT *p*-values suggested improved statistical fit with increasing model complexity, although parallel responses in the final 6-profile solution and the corresponding drop in entropy indicated a potential overextraction. Given the identification of a conceptually distinct subpopulation in the 5-profile model combined with stable values for entropy, this model was selected as the preferred solution to these data.

**FIGURE 2:**
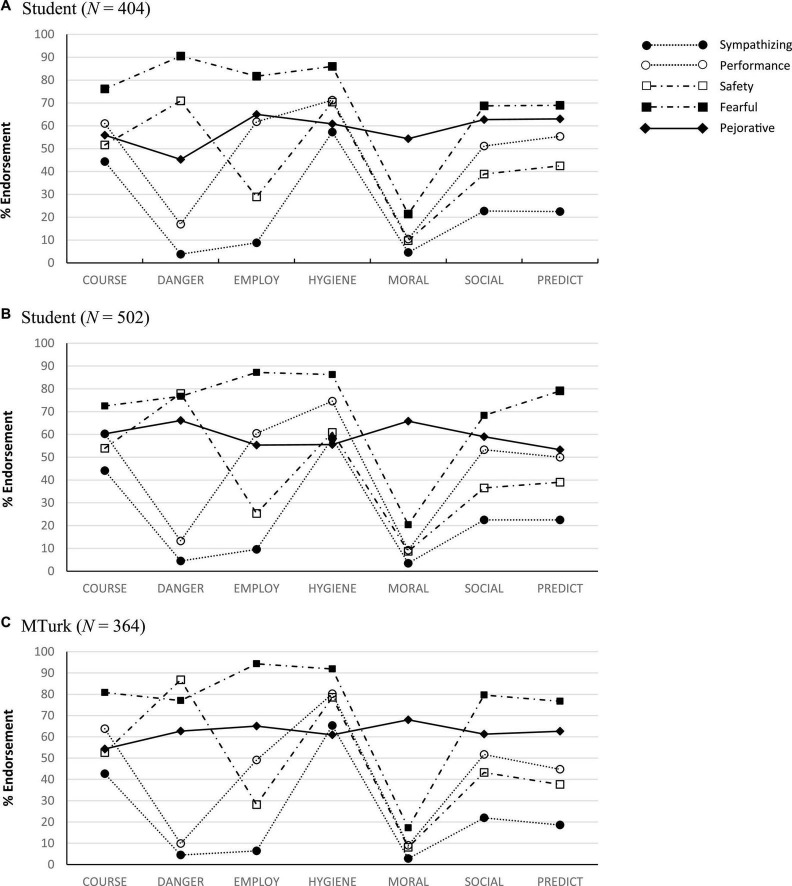
Latent profiles for Study 1 **(A)**, Study 2 **(B)**, and Study 3 **(C)**.

Subgroups for the 5-profile model are presented in Figure 2A with means and confidence intervals for domain scores reported in Table 2. The first profile in this solution (*Sympathizing*) captured the largest proportion of respondents (62.4%). Individuals in this subgroup reported the lowest levels of stereotype endorsement, although results did indicate elevated beliefs for course and mental hygiene domains as well as modest agreement with statements related to social concerns and predictability. By contrast, the *Pejorative* profile (5.7%) was marked by consistent endorsement across all stereotype domains. Agreement with course and mental hygiene beliefs was comparable to that observed in the Sympathizing profile (see Supplementary Figure A for a comparison of profile means and confidence bounds). However, scores for dangerousness, employability, social concerns, and predictability were noticeably higher with elevations in Moralizing serving as the defining characteristic of the group. An additional high-stereotype profile (*Fearful*; 7.2%) evidenced the largest absolute scores for all belief domains with the exception of moralizing. Agreement with course, dangerousness, and mental hygiene items were the highest of any class. Elevations in employment, social concerns, and predictability were commensurate with those in the Pejorative subgroup. Of the two intermediate profiles, respondents were distinguished primarily by stereotypes involving beliefs regarding dangerousness and employability. *Safety-Focused* participants (9.4%) endorsed dangerousness stereotypes at levels second only to those in the Fearful profile. *Performance-Focused* individuals (18.3%), by contrast, reported employability concerns comparable to those noted in the Fearful and Pejorative subgroups. Safety- and Performance-Focused profiles were similar in stereotype endorsement across all other domains.

**TABLE 2:** Domain scores and 95% confidence intervals for Study 1, Study 2, and Study 3^a^.

	Course	Danger	Employ	Hygiene	Moral	Social	Predict
**Study 1**							
SYMP (*n* = 252)	44.4 [41.1, 47.6]	3.8 [2.3, 5.4]	8.8 [6.4, 11.3]	57.2 [54.4, 60.1]	4.6 [3.3, 5.9]	22.7 [20.3, 25.1]	22.5 [18.7, 25.4]
PERF (*n* = 62)	61.0 [56.0, 66.0]	16.9 [11.2, 22.6]	61.8 [36.4, 87.3]	71.2 [65.7, 76.7]	10.5 [6.1, 14.9]	51.1 [41.4, 60.8]	55.4 [48.4, 62.4]
SAFE (*n* = 38)	51.6 [41.7, 61.6]	70.9 [61.3, 80.5]	28.9 [18.2, 39.6]	70.3 [62.3, 78.4]	9.7 [1.1, 18.4]	38.9 [32.8, 45.0]	42.5 [30.0, 54.9]
FEAR (*n* = 29)	76.1 [67.3, 84.9]	90.5 [82.2, 98.9]	81.7 [56.6, 100.0]	86.0 [79.6, 91.3]	21.4 [14.8, 28.0]	68.7 [53.5, 84.0]	69.0 [62.5, 75.4]
PEJOR (*n* = 23)	55.9 [45.3, 66.5]	45.3 [29.3, 61.2]	65.0 [37.8, 92.3]	60.9 [45.4, 76.3]	54.4 [45.1, 63.6]	62.7 [49.4, 75.9]	63.0 [52.0, 74.0]
**Study 2**							
SYMP (*n* = 335)	44.1 [41.6, 46.6]	4.5 [3.1, 5.9]	9.6 [7.4, 11.8]	58.3 [55.8, 60.7]	3.5 [2.6, 4.3]	22.5 [20.2, 24.8]	22.5 [20.0, 25.0]
PERF (*n* = 74)	60.3 [55.2, 65.4]	13.3 [8.5, 18.0]	60.5 [41.2, 79.7]	74.6 [70.3, 78.9]	9.2 [5.4, 13.0]	53.3 [47.4, 59.1]	50.0 [45.2, 54.7]
SAFE (*n* = 46)	53.9 [48.0, 59.8]	78.0 [71.7, 84.3]	25.3 [16.9, 33.7]	60.9 [54.5, 67.4]	8.7 [5.3, 12.0]	36.5 [31.3, 41.6]	39.0 [33.3, 44.7]
FEAR (*n* = 32)	72.5 [66.8, 78.3]	76.7 [66.6, 86.7]	87.2 [77.6, 96.7]	86.3 [81.3, 91.3]	20.4 [13.9, 26.9]	68.3 [63.4, 73.2]	79.1 [71.2, 87.0]
PEJOR (*n* = 15)	60.3 [48.9, 71.7]	66.1 [46.6, 85.6]	55.3 [34.7, 75.9]	55.6 [41.1, 70.2]	65.8 [50.9, 80.6]	59.0 [44.3, 73.6]	53.3 [40.0, 66.5]
**Study 3**							
SYMP (*n* = 210)	42.6 [39.1, 46.0]	4.5 [2.6, 6.5]	6.4 [4.1, 8.6]	65.3 [61.7, 68.9]	2.8 [1.8, 3.8]	21.9 [19.6, 24.3]	18.6 [16.6, 20.6]
PERF (*n* = 70)	63.8 [60.2, 67.5]	9.8 [5.9, 13.8]	49.1 [37.2, 61.0]	80.2 [75.3, 85.1]	9.0 [5.7, 12.3]	51.7 [47.1, 56.2]	44.7 [39.1, 50.4]
SAFE (*n* = 42)	52.5 [46.0, 58.9]	86.8 [81.0, 92.6]	28.1 [19.5, 36.7]	78.5 [74.0, 83.0]	8.1 [4.9, 11.4]	43.2 [36.5, 49.9]	37.6 [32.6, 42.7]
FEAR (*n* = 26)	80.8 [76.1, 85.5]	77.0 [64.4, 89.6]	94.3 [88.5, 95.2]	91.9 [88.5, 95.2]	17.2 [11.2, 23.2]	79.6 [73.9, 85.3]	76.7 [69.9, 83.6]
PEJOR (*n* = 16)	54.3 [44.5, 64.2]	62.7 [45.8, 79.7]	65.0 [48.7, 81.4]	60.9 [50.6, 71.2]	68.0 [57.8, 78.3]	61.3 [50.8, 71.8]	62.6 [50.8, 74.4]

SYMP, sympathizing profile; PERF, performance-focused profile; SAFE, safety-focused profile; FEAR, fearful profile; PEJOR, pejorative profile.

^a^Means and interval estimates based on latent profile analysis in MPlus.

#### 2.2.3. Group comparisons

Associations of group membership with external variables were examined in a series of chi square and ANOVA models. Analysis indicated modest evidence of sex differences in profile membership (*p* = 0.024, *V* = 0.167; *s* = 5, BFB = 4.13) with men overrepresented among Pejorative respondents (*z*_adj_ = 3.14; see Table 3). Results, however, failed to support systematic associations with participant trauma (*p* = 0.967, *V* = 0.037), prior treatment (*p* = 0.783, *V* = 0.066), or reported exposure in a close friend or family member (*p* = 0.580, *V* = 0.084). Data also failed to provide evidence for associations with social desirability (*p* = 0.289, η^2^ = 0.012) or positive affect (*p* = 0.894, η^2^ = 0.003). A small magnitude effect was observed for differences in negative affectivity across belief profiles (*p* = 0.007, η^2^ = 0.065; *s* = 7, BFB = 11.24). Games-Howell follow-up tests offered moderate support for greater levels of negative affect in Pejorative (*M* = 20.83, SD = 9.01) versus Sympathizing respondents (*M* = 14.96, SD = 5.37; *p* = 0.038, *d* = 1.02; *s* = 5, BFB = 2.96). No additional pairwise effects were noted in these analyses (all *p* ≥ 0.098).

**TABLE 3:** Covariate scores for Study 1, Study 2, and Study 3.

	Sex	PT^a^	Tx	ffPT	MCSD^b^	PANPOS	PANNEG	MSPSS	CAMIauth	CAMIben	CAMIrest	CAMImhi
**Study 1**												
1. SYMP	73.8	51.2	24.2	41.7	16.6 (5.1)	28.1 (8.2)	15.0 (5.4)					
2. PERF	77.4	54.8	21.0	45.2	16.1 (5.2)	28.6 (8.8)	15.6 (5.4)					
3. SAFE	73.7	52.6	21.1	44.7	15.4 (5.0)	27.1 (9.8)	18.0 (7.4)	–	–	–	–	–
4. FEAR	65.5	55.2	24.1	44.8	14.8 (5.1)	29.0 (8.5)	16.6 (5.9)	–	–	–	–	–
5. PEJOR	43.5	34.8	13.0	26.1	15.3 (4.7)	28.2 (7.2)	20.8 (9.0)	–	–	–	–	–
**Study 2**												
1. SYMP	69.0	60.6	26.3	40.6	6.4 (2.7)	29.5 (7.9)	15.9 (5.9)	5.6 (1.3)	12.6 (5.0)	28.9 (5.7)	12.0 (5.2)	26.7 (5.8)
2. PERF	73.9	63.0	23.9	39.1	5.5 (2.3)	28.4 (7.9)	17.2 (6.1)	5.5 (1.2)	14.1 (5.1)	27.7 (5.5)	14.0 (5.1)	25.2 (5.7)
3. SAFE	66.2	50.0	27.0	47.3	6.1 (2.7)	29.0 (8.5)	17.1 (5.6)	5.7 (1.1)	14.1 (4.9)	27.8 (6.2)	12.9 (5.6)	25.3 (6.4)
4. FEAR	71.9	59.4	21.9	46.9	6.2 (2.7)	29.0 (8.5)	17.1 (5.6)	5.7 (1.1)	14.1 (4.9)	27.8 (6.2)	12.9 (5.6)	25.3 (6.4)
5. PEJOR	53.3	80.0	6.7	40.0	6.1 (2.6)	34.3 (5.0)	17.0 (4.8)	5.6 (1.2)	18.3 (4.8)	23.7 (5.5)	16.3 (3.9)	22.0 (4.4)
**Study 3**												
1. SYMP	65.7	36.7	52.4	57.1	5.8 (3.5)	27.6 (8.3)	12.6 (4.7)	5.4 (1.2)	10.1 (5.1)	32.1 (5.6)	9.9 (5.9)	29.0 (6.9)
2. PERF	77.1	34.3	48.6	52.9	5.5 (2.9)	27.8 (8.1)	14.2 (6.1)	5.3 (1.2)	12.0 (5.2)	30.4 (6.5)	11.6 (5.4)	27.4 (5.8)
3. SAFE	64.3	23.8	47.6	45.2	4.7 (2.8)	31.1 (7.4)	13.3 (5.3)	5.7 (1.0)	12.0 (5.5)	31.6 (5.4)	12.4 (5.8)	26.5 (7.3)
4. FEAR	42.3	3.9	38.5	42.3	4.9 (2.6)	25.7 (7.7)	14.5 (7.6)	4.9 (1.7)	14.1 (5.2)	28.6 (6.7)	14.5 (5.9)	25.0 (7.3)
5. PEJOR	43.8	25.0	18.8	25.0	6.5 (3.2)	29.1 (9.3)	15.8 (6.9)	5.0 (1.4)	21.1 (3.8)	21.7 (5.2)	20.8 (4.6)	21.2 (6.2)

SYMP, sympathizing; PERF, performance-focused; SAFE, safety-focused; FEAR, fearful; PEJOR, pejorative; Sex, % female; PT, % probable trauma; Tx, % prior mental health treatment; ffPT, % trauma exposed friends/family; MCDS, Marlowe–Crowne social desirability scale; PANAS, positive and negative affective schedule; MSPSS, multidimensional scale of perceived social support; CAMI, community attitudes towards mental illness. ^a^History of probable trauma assessed with a single item in Study 3. ^b^Studies 2 and 3 assessed using the 13-item Marlowe-Crowne short-form.

## 3. Study 2

Analyses in Study 1 identified five distinct profiles of stereotype endorsement characterized by beliefs about the impact of trauma across course, dangerousness, employability, mental hygiene, moralizing, social concern, and predictability domains. The aims of Study 2 were to determine the extent to which belief profiles and observed associations with background characteristics, social desirability, and state affect would replicate within an independent sample of university students (*N* = 502). Analyses also included a series of linear contrasts testing expected differences in (a) social support and (b) global perceptions of mental illness based on profile conceptualizations developed in Study 1.

### 3.1. Methods

#### 3.1.1. Participants

Participants were university students (*N* = 502) completing an online, mass-testing procedure for psychological research. No exclusion criteria were implemented aside from restricting participation to students who were not involved in the previous study.^3^ Respondents identified predominantly as female (68.7%) and White/Non-Hispanic (77.3%). Mean age of the sample was 19.9 years (SD = 3.4). Roughly 35% of the sample reported at least one event consistent with potential Criterion-A trauma based on responses to the ESP (disaster = 3.2%; fire = 2.6%; traffic accidents = 18.3%; physical assault = 4.4%; sexual violence = 13.8%; other trauma = 6.2%). Full sample characteristics are available in Table 1.

#### 3.1.2. Measures

All measures described in Study 1 were administered in Study 2 except for the replacement of the MCSDS with a short-form version. Two additional scales were included in the test battery for this sample.

##### 3.1.2.1. Marlowe–Crowne social desirability scale-short form

The MCSD-SF is an abbreviated, 13-item version of the MCSDS (Reynolds, 1982). Previous research has demonstrated strong correlations of the MCSD-SF with the original 33-item scale (*r* = 0.93; Reynolds, 1982). Internal consistency in the current sample was modest (α = 0.65).

##### 3.1.2.2. Multidimensional scale of perceived social support

The MSPSS (Zimet et al., 1988) is a 12-item scale developed to assess perceptions of social support. Statements indicative of perceived support are rated on a 7-point Likert scale (1 = *very strongly disagree*; 7 = *very strongly agree*). Total MSPSS scores are calculated as the mean of completed items with higher scores indicating greater levels of perceived support. Evidence for the factorial validity of the MSPSS has been observed in both student and psychiatric samples (e.g., Clara et al., 2003). The internal consistency of items was excellent in these data (α = 0.95).

##### 3.1.2.3. Community attitudes towards mental illness

The CAMI (Taylor and Dear, 1981) is a 40-item measure of public attitudes toward individuals with mental health concerns. Ratings are made on a 5-point Likert scale (1 = *strongly agree*, 5 = *strongly disagree*) and summed to produce four subscales. *Authoritarianism* is intended to measure the perception of individuals with mental health difficulties as an inferior class requiring coercive handling. *Benevolence* captures sympathetic and paternalistic attitudes toward individuals with mental illness and a belief in the responsibility of communities to assist those with psychological difficulties. *Social Restrictiveness* refers to the belief that people with mental health concerns are dangerous and unpredictable and should be avoided. *Community Mental Health Ideology (CMHI)* corresponds to a belief in the value of mental health facilities/services in the local community and a commitment to deinstitutionalized care. Scores from the current sample provided strong estimates of internal consistency for authoritarianism (α = 0.76), benevolence (α = 0.87), social restrictiveness (α = 0.83), and CMHI (α = 0.90) scales.

#### 3.1.3. Analytic plan

A 5-profile LPA was estimated in MPlus using procedures identical to those in Study 1. Associations of profile membership with variables captured in the previous analyses (i.e., sex, probable Criterion-A exposure, treatment history, reported friend/family trauma, state mood, social desirability) were assessed to examine the stability of estimates across independent samples. ANOVA models were also used to evaluate a series of *a priori*, linear contrasts for MSPSS and CAMI scales based on conceptualizations of profiles derived from Study 1. Specifically:

• Respondents in the Pejorative and Fearful profiles were expected to report lower levels of perceived support relative to other subgroups. Uniformly high endorsement of stereotyped beliefs and – for the Pejorative profile – overtly prejudiced statements were hypothesized to reflect low levels of agreeableness that could impact more general indices of social functioning.
• Pejorative and Fearful respondents were also expected to report higher CAMI authoritarianism scores as compared to other subgroups. Again, uniformly high endorsement of stereotyped beliefs in these profiles was believed to be consistent with generalized negative attitudes toward individuals with mental health concerns.
• Sympathizing respondents were expected to demonstrate higher scores on CAMI benevolence than other profiles given low levels of stereotype endorsement outside of potentially well-intended beliefs regarding course and mental hygiene.
• Pejorative, Fearful, and Safety-Focused profiles were expected to report higher levels of CAMI restriction as compared to other groups. For Safety-Focused respondents, specific elevations in dangerousness stereotypes were expected to result in increased desire for social distance and heightened perceptions of individuals with mental health concerns as a potential threat.
• Members of the Sympathizing profile were expected to demonstrate higher scores on CAMI CMHI than other subgroups given evidence of low stereotype endorsement across dangerousness, employability, social concern, and predictability domains.

Linear contrasts and 95% confidence bounds around mean differences (ψ) were calculated in Stata.^4^ Effect sizes for specific contrasts were standardized using the square root of the MS_error_ from the omnibus ANOVA.

### 3.2. Results

#### 3.2.1. Stereotype endorsement

Item-level endorsement of individual beliefs was nearly identical to rates observed in Study 1 (see Figure 1). Results suggest that the acknowledgement of specific, trauma-related stereotypes occurs at frequencies that are replicable in the student population from which this sample was drawn. Rates of agreement with individual TBS items are provided in Supplementary Table A.

#### 3.2.2. LPA

Estimates from the 5-profile LPA extracted from this sample (AIC = 30,516.2; BIC = 30710.3; BLRT *p* < 0.001; entropy = 0.94) replicated subgroups observed in Study 1 (see Figure 2).^5^ Representation across individual profiles was identical to patterns noted in the previous study, with the majority of respondents falling in the Sympathizing profile (66.7%) followed by Performance-Focused (14.7%), Safety-Focused (9.2%), Fearful (5.8%), and Pejorative (4.6%) groups. Domain scores and confidence bounds for Study 2 profiles are available in Table 2. Differences in profile scores across stereotype domains are represented graphically in Supplementary Figure B.

#### 3.2.3. Group comparisons

Comparisons in these data failed to support differences in profile membership with respect to sex (*p* = 0.577, *V* = 0.076), participant trauma (*p* = 0.281, *V* = 0.100), prior psychological treatment (*p* = 0.508, *V* = 0.081), or reported trauma in friends or family members (*p* = 0.822, *V* = 0.055). Results also failed to provide compelling evidence for differences in social desirability (*p* = 0.304, η^2^ = 0.010) or positive (*p* = 0.139, η^2^ = 0.033) and negative affect (*p* = 0.052, η^2^ = 0.041) across profiles.

Linear contrasts provided mixed support for *a priori* hypotheses. Contrary to expectation, respondents reported similar levels of perceived support across stereotype profiles (*p* = 0.897, η^2^ = 0.002). Results did, however, provide evidence for differences in CAMI authoritarianism (*p* = 3.00E-8, η^2^ = 0.079; *s* = 25, BFB = 7.08E + 5), benevolence (*p* = 4.51E-4, η^2^ = 0.040; *s* = 11, BFB = 105.9), social restrictiveness (*p* = 1.67E-5, η^2^ = 0.054; *s* = 16, BFB = 1,999.2), and CMHI (*p* = 0.001, η^2^ = 0.035; *s* = 10, BFB = 43.1) scores. Consistent with hypotheses, the combination of Pejorative and Fearful respondents reported greater levels of CAMI authoritarianism relative to other subgroups (ψ = 4.05, CI_95%_ [2.39, 5.70], *p* = 2.10E-6, *d* = 0.81; *s* = 19, BFB = 13,407.1). Participants in the Sympathizing profile, by contrast, evidenced higher scores on CAMI benevolence as compared to the combination of other groups (ψ = 2.64, CI_95%_ [1.45, 3.82], *p* = 1.45E-5, *d* = 0.47; *s* = 16, BFB = 2,279.7). Pejorative, Fearful, and Safety-Focused respondents demonstrated greater levels of CAMI social restrictiveness relative to the collection of Performance-Focused and Sympathizing profiles (ψ = 2.99, CI_95%_ [1.61, 4.37], *p* = 2.34E-5, *d* = 0.56; *s* = 15, BFB = 1,473.1). Finally, Sympathizing respondents evidenced greater CMHI scores compared to the combination of all other groups (ψ = 2.63, CI_95%_ [1.40, 3.85], *p* = 3.03E-5, *d* = 0.45; *s* = 15, BFB = 1,166.4). Descriptive statistics for external scales for all profiles are provided in Table 3. Means and interval estimates for CAMI scores are plotted in Supplementary Figure D.

## 4. Study 3

Results of Study 2 provided compelling support for the replicability of stereotype profiles in an independent sample drawn from the same population as the initial study. Data also offered preliminary evidence for predicted associations between profile membership and global attitudes toward persons with mental health concerns. The aims of Study 3 were to determine the extent to which effects noted in the first two samples generalize to respondents sampled from a separate, non-student population.

### 4.1. Methods

#### 4.1.1. Participants

Participants included US residents (*N* = 364) recruited through Amazon’s Mechanical Turk (MTurk), an internet-based marketplace where users complete online tasks for monetary compensation. Participation was restricted to US residents, but no other exclusion criteria were implemented for the study. Respondents continued to identify predominantly as female (65.1%) and White/Non-Hispanic (76.1%). Participants were markedly older (*M* = 35.9, SD = 3.4) than those included in Study 1 and 2, although it is relevant to note that nearly all respondents in the MTurk sample reported some level of higher education (95.1% completing some college or greater). Participants varied by geographic region and half of respondents reported full-time employment (51.6%). Nearly a quarter (22.7%) identified current student status. Full demographic information is provided in Table 1.

#### 4.1.2. Measures

Assessment instruments were identical to those in Study 2 except that exposure to probable trauma in this sample was assessed using a single dichotomous (0 = *no*, 1 = *yes*) item (*Have you personally experienced a significant trauma?*). Those reporting a previous exposure were asked to identify the type of event (*If YES, what was the specific event [e.g., accident, sexual assault, combat exposure, etc.]?*). Based on this single-item screener, 38.5% of the sample identified histories of potential trauma (disaster = 0.6%; fire = 0.3%; traffic accidents = 6.6%; physical assault = 9.9%; sexual violence = 14.8%; other trauma = 5.8%).

#### 4.1.3. Analytic plan

The analytic approach for Study 3 was identical to that presented in Study 2. Hypotheses for linear contrasts in the previous study were retained given efforts to (a) replicate effects from Study 2 in participants from a distinct population and (b) avoid altering predictions based on possible sample-specific patterns observed in the previous set.

### 4.2. Results

#### 4.2.1. Stereotype endorsement

Results again demonstrated a high degree of consistency in the endorsement of item-level stereotypes relative to data collected in Studies 1 and 2 (see Figure 1). Results suggest that the prevalence of trauma-related stereotypes generalizes to populations beyond current university students. Rates of agreement with specific TBS items in Study 3 are provided in Supplementary Table A.

#### 4.2.2. LPA

Estimates from the 5-profile LPA (AIC = 22,223.8; BIC = 22,403.0; BLRT *p* < 0.001; entropy = 0.95) replicated subgroups previously observed in university undergraduates (see Figure 2).^6^ Representation of individual profiles was similar to that of previous analyses with the majority of respondents falling in the Sympathizing profile (57.7%) followed by Performance-Focused (19.2%), Safety-Focused (11.5%), Fearful (7.1%), and Pejorative (4.4%) classes. Domain scores and corresponding confidence bounds for Study 3 profiles are available in Table 2. A comparison of profile means and associated confidence intervals for individual stereotype domains is available in Supplementary Figure C.

#### 4.3.3. Group comparisons

Associations with respondent characteristics indicated potential sex differences across stereotype profiles (*p* = 0.007, *V* = 0.197; *s* = 7, BFB = 10.59) with women overrepresented among Performance-Focused respondents (*z*_adj_ = 2.22) and men overrepresented in the Fearful profile (*z*_adj_ = 2.63; see Table 3). Profile membership was also associated with probable trauma in this sample (*p* = 4.75E-4, *V* = 0.236; *s* = 11, BFB = 101.2). Specifically, individuals reporting likely exposure were more strongly represented among Sympathizing respondents (*z*_adj_ = 3.32) and underrepresented in the Fearful profile (*z*_adj_ = −3.79). Analyses failed to provide evidence for associations of profile membership with prior psychological treatment (*p* = 0.092, *V* = 0.149) or reported trauma in close friends or family (*p* = 0.071, *V* = 0.154). Data also failed to support differences with respect to social desirability (*p* = 0.182, η^2^ = 0.017) or positive affect (*p* = 0.067, η^2^ = 0.024). Results did offer some evidence for differences in negative affectivity across profiles (*p* = 0.035, η^2^ = 0.028; *s* = 5, BFB = 3.8). Games-Howell *post hoc* tests, however, did not indicate differences meeting traditional benchmarks for interpretation (all *p* ≥ 0.247).

The pattern of results for linear contrasts were identical to those in Study 2. Similar levels of perceived support were observed across stereotype profiles (*p* = 0.069, η^2^ = 0.024). However, data offered compelling evidence for differences in CAMI authoritarianism (*p* = 5.51E-15, η^2^ = 0.183; *s* = 47, BFB = 2.03E + 12), benevolence (*p* = 4.67E-10, η^2^ = 0.128; *s* = 31, BFB = 3.67E + 7), social restrictiveness (*p* = 1.45E-12, η^2^ = 0.157; *s* = 39, BFB = 9.30E + 9), and CMHI (*p* = 1.73E-5, η^2^ = 0.073; *s* = 16, BFB = 1,937.91). As before, the combination of Pejorative and Fearful respondents reported greater levels of CAMI authoritarianism relative to other subgroups (ψ = 6.23, CI_95%_ [4.49, 7.97], *p* = 9.83E-12, *d* = 1.22; *s* = 37, BFB = 1.48E + 9). Participants in the Sympathizing profile, by contrast, evidenced higher benevolence scores than the combination of other profiles (ψ = 3.96, CI_95%_ [2.63, 5.30], *p* = 1.11E-8, *d* = 0.68; *s* = 26, BFB = 1.81E + 6). Pejorative, Fearful, and Safety-Focused respondents demonstrated greater levels of CAMI social restrictiveness relative to the collection of Performance-Focused and Sympathizing classes (ψ = 5.17, CI_95%_ [3.63, 6.71], *p* = 1.39E-10, *d* = 0.90; *s* = 32, BFB = 1.16E + 8). Finally, Sympathizing respondents evidenced greater CMHI scores as compared to aggregate scores from all other groups (ψ = 3.97, CI_95%_ [2.44, 5.51], *p* = 6.08E-7, *d* = 0.59; *s* = 20, BFB = 4.23E + 4). Descriptive statistics for external scales are provided in Table 3. Profile means and interval estimates for CAMI scores across groups are plotted in Supplementary Figure D.

## 5. Discussion

The aims of the current, multi-study project were to (a) provide an initial assessment of the degree to which trauma-related stereotypes are endorsed in members of the general community, (b) examine person-level variability in stereotype endorsement to identify replicable patterns of held beliefs, and (c) evaluate the associations of belief profiles with person-level characteristics and general perspectives on mental illness. Data collected for this research confirm that members of the general public *do* endorse a range of stereotyped beliefs involving the chronicity of post-trauma responses; the inherent dangerousness of survivors; lowered expectations in employment settings; assumed impairment in social domains; blame and suspicion of those demonstrating post-trauma reactions; concerns related to the predictability and emotional stability of survivors; and a perceived need for formal psychological intervention in response to exposure. Also telling is the frequency at which stereotypes were endorsed across samples. For example, 40–47% of respondents agreed with the statement, *“People exposed to serious trauma are damaged.”* Nearly a quarter of those surveyed agreed with the statement, *“People exposed to serious trauma often become dangerous.”* A similar proportion indicated, *“It is difficult to work with someone who has experienced serious trauma.”* Over 80% of those sampled disagreed with the statement, *“People exposed to serious trauma generally do not need therapy.”* Similarly, 38–44% disagreed with the statement, *“People exposed to serious trauma have healthy family relationships.”* Approximately 90% failed to endorse the item, *“People exposed to serious trauma are as psychologically stable as they were before.”* Even moralizing beliefs with obvious pejorative content were endorsed at surprising levels, including the identification of survivors as emotionally weak (16.8–24.3%), needy (13.2–20.1%), and as making excuses for their behavior (7.7–12.6%). Results suggest that survivor-reported mistrust of others and perceptions of blame, judgment, and discrimination (e.g., Clapp et al., 2014, 2022; Relyea and Ullman, 2015; Dardis et al., 2018) may – in part – be founded.

Patterns of stereotype endorsement were also remarkably consistent both within and across populations sampled for this research. Item-level responses were nearly identical for undergraduate and MTurk volunteers. Stereotype profiles based on aggregate domain scores also replicated across samples. Fearful participants were characterized by the highest level of absolute endorsement across stereotype domains, with the exception of moralizing beliefs. Results indicate a subset of the public that is less likely to identify survivors as weak, needy, or making excuses for their behavior but quite likely to agree that the consequences of trauma are permanent. Fearful respondents are likely to believe that survivors are inherently dangerous, unpredictable, and impaired across social and occupational domains and that formal treatment is needed following any exposure. Pejorative respondents also demonstrated uniform elevations in most stereotype domains, with the endorsement of moralizing items serving as the defining feature of this profile. Interestingly, beliefs corresponding to mental health hygiene were the lowest or among the lowest of any profile across samples; indicating general assumptions of permanence, dangerousness, impairment, and moral failings, but relatively low perceptions of survivors’ need for intervention. Participants in the Safety-Focused profile demonstrated intermediate levels of stereotype endorsement with the exception of elevations in perceived dangerousness. Performance-Focused respondents evidenced a similar pattern, substituting elevations in perceived dangerousness with elevated presumptions of occupational impairment. Social-cognitive models of mental health stigma (e.g., Corrigan et al., 2003) outline clear pathways by which stereotypes documented in this research may translate into prejudicial behaviors noted in the larger trauma literature (e.g., Bromfield et al., 1988; Ullman, 1999; Purtle et al., 2016; Dworkin et al., 2019).

The Sympathizing profile is arguably the most interesting class extracted from these analyses. Sympathizing respondents evidenced the lowest levels of stereotype endorsement of any group, but continued to demonstrate relative elevations in course and mental health hygiene, along with modest agreement in social concern and predictability domains. This pattern of beliefs – while favorable relative to other profiles – could easily translate into inadvertent patterns of well-intended, but unhelpful, behavior. Elevations in the expected chronicity of trauma reactions among Sympathizing respondents can be understood as a reflection of strong beliefs in the general public about the association of psychological stress and mental illness (Schomerus et al., 2012). These beliefs are likely to elevate the endorsement of hygiene items as well as general positive attitudes toward the utility of mental health treatment (Parcesepe and Cabassa, 2013). However, while this particular orientation could facilitate support, it may also lead to the pathologizing of normative stress reactions and/or the elicitation of controlling and paternalistic responses noted in previous studies (e.g., Ullman and Filipas, 2001; Holguin and Hansen, 2003; Relyea and Ullman, 2015; Dworkin et al., 2019). These data, combined with existing work on the complexities of post-trauma support, highlight the importance of continued research on survivor needs and support member assumptions and the ways in which these factors interact to influence recovery.

The associations of belief profiles with external variables were generally consistent with hypotheses. Results failed to provide evidence for systematic differences in perceived support across stereotype groups, suggesting that prejudicial beliefs about trauma and its consequences are not linked to broad deficits in relationships with close others. Analyses did, however, support convergent associations with more general perspectives on mental illness. *A priori* contrasts confirmed small to moderate increases in reported benevolence and preference for deinstitutionalized mental health care in Sympathizing respondents relative to the combination of other profiles. Fearful and Pejorative participants evidenced large magnitude elevations in authoritarian attitudes toward individuals with mental health difficulties relative to Performance-Focused, Safety-Focused, and Sympathetic classes. Fearful, Pejorative, and Safety-Focused profiles also demonstrated moderate to large increases in preferences for social restriction as compared to other groups. The expected correspondence with general perspectives on mental health bolsters confidence in the interpretation of profiles extracted from these data. It is worth noting that Sympathizing, Performance-Focused, Safety-Focused, Fearful, and Pejorative subgroups demonstrated ordered increases/decreases in CAMI scores within both undergraduate and MTurk samples (see Supplementary Figure D). These patterns, however, were not reflected as sequential elevations in the endorsement of trauma-related stereotypes measured in this research. Results highlight calls from investigators in the larger stigma literature for more nuanced approaches to the assessment of mental health stereotypes, considering heterogeneity both within and across groups as well as the ways in which patterns of problem/condition-specific beliefs may impact targeted populations (e.g., Hinshaw and Stier, 2008; Abdullah and Brown, 2011; Angermeyer and Schomerus, 2017).

Preliminary analyses suggest that confounding factors do not easily account for patterns noted in this research. Data provide little evidence for the potential impact of state positive affect or social desirability on stereotype endorsement. Results did demonstrate some moderate support for possible associations of profile with negative affectivity although specific differences failed to replicate across samples. The association of profiles with background characteristics was similarly mixed. Sex differences were observed in two of the three samples, but effects were small and the specific pattern of over/under representation across groups was inconsistent. Treatment history and the identification of trauma exposure in close friends and/or family was unrelated to stereotype group. Participant report of personal trauma exposure was associated with a greater likelihood of classification as Sympathizing in the MTurk sample, but corresponding effects were not observed in undergraduate respondents. Results suggest that the development of lay beliefs about mental health difficulties is complex (e.g., Haslam, 2005) and that trauma exposure – by personal experience or through association with close others – may not directly inform stereotypes endorsed in the general community.

### 5.1. Limitations and future directions

Interpretation of these data should be made within the context of the relative strengths and limitations of the project as a whole. This research is the first to conduct a broad-based assessment of public stereotypes about trauma exposure and its potential impact on survivors. The project is also the first to model unobserved heterogeneity in stereotype endorsement to explore patterns of stigmatizing beliefs in those who may serve as friends, family, and/or support members to trauma-exposed individuals. The incorporation of multiple, large, independent samples facilitated the internal replication of stereotype profiles as well as tests of associations with person-level characteristics. The collection of data from two distinct respondent populations also provides initial support for the generalizability of observed effects.

It is important, however, to recognize that the endorsement of trauma-related stereotypes in this research overwhelmingly reflect views in a subset of White, U.S., majority culture. Abdullah and Brown (2011) provide the most comprehensive review of ethnocultural influences on mental health stigma to date. These authors note that all forms of stigma are inherently bound by culture, meaning that overall levels and specific profiles of trauma-related stereotypes could fail to generalize in other communities, both within and outside the U.S. Although results of the current project offer support for the potential replicability of results in young- to middle-aged, educated, White/Non-Hispanic adults, the exploration of stigmatizing beliefs in more diverse subsets of the population will be critical to understanding specific pressures on survivors in those communities. It is also worth noting that women were overrepresented as respondents in all three samples, with a higher proportion of MTurk participants identifying as female relative to estimates of workers as a whole. While respondent sex failed to demonstrate systematic associations with stereotype profiles in these data, the intersection of gender and other cultural factors on attitudes toward survivors warrants continued attention in future research.

The precision of some estimates are also influenced by the low proportion of respondents classified into profiles marked by high levels of stereotype endorsement. Again, the current project benefited from the use of large samples which were able to capture various high-stigma groups. Replication across independent studies also strengthens conclusions regarding the stability of extracted profiles. The limited number of participants in the Fearful and Pejorative groups, however, does introduce additional uncertainty in tests of associated characteristics. Future studies will need to continue to utilize large-sample methods to assure the representation of classes that may display particularly toxic responses toward survivors.

Finally, observed stigma profiles were based on responses to a novel, descriptive survey developed specifically for the purposes of this research. Items for individual beliefs and larger stereotype domains were drawn from established literature on mental health stigma (e.g., Link and Phelan, 2001; Hinshaw and Stier, 2008) as well as research exploring public and support-member reactions in various survivor populations (e.g., Currier et al., 2013; Tener and Murphy, 2015; Dworkin et al., 2019). Although the assessment of specific domains was broad, it is by no means inclusive of all negative beliefs members of the public may have about trauma and its consequences. The current series of studies offers preliminary support for the high frequency of trauma stereotype endorsement and for the utility of the TBS in capturing problematic beliefs across common stigma domains. However, the replication of effects in other samples; the assessment of potentially overlooked forms of stigmatization in diverse survivor groups; and an examination of the extent to which the endorsement of trauma stereotypes correspond to overt, prejudicial behavior are all important avenues for continued research.

### 5.2. Conclusion

Deficits in post-trauma support and the disruption of interpersonal processes in survivor groups have been noted for decades. Research, understandably, has tended to focus on symptoms, perceptions, and cognitive biases in those exposed to traumatic events while placing less emphasis on potential support members and individuals in survivors’ larger communities. The current data provide compelling evidence for the widespread endorsement of trauma-related stereotypes and for distinct patterns of beliefs that may contribute to specific points of prejudice (e.g., employment, relationships, health care). Understanding beliefs held by the general community and how these may impact support and other opportunities will be an important consideration in continued research on post-trauma functioning.

## Data availability statement

The datasets presented in this study can be found in online repositories. The names of the repository/repositories and accession number(s) can be found below: Open Science Framework (https://osf.io/cjhyd).

## Ethics statement

The studies involving human participants were reviewed and approved by University of Wyoming Human Subjects Institutional Review Board. The patients/participants provided their written informed consent to participate in this study.

## Author contributions

JC, AS, and SF: conceptualization. JC: methodology, formal analysis, and writing – original draft preparation. JC, AS, LE, RK, and AB: investigation. AS, SF, LE, RK, and AB: writing – review and editing. All authors contributed to the article and approved the submitted version.

## Footnotes

1 Although participants were students actively enrolled in undergraduate psychology courses, only a minority of respondents were declared Psychology majors given broad enrollment in psychology coursework at the sponsoring institution, particularly in large-section 1000 and 2000 level courses. As such, participants in this research are composed of students from diverse programs of study.

2 MPlus does not calculate exact *p*-values for BLRT beyond the 0.0001 level.

3 Person identifiers included in the collection of basic demographic information were used to identify and exclude any individuals having previously participated in Study 1.

4 Linear contrasts were developed to focus analyses on specific, *a priori* predictions based on the conceptualization of profiles derived in Study 1. However, means and standard deviations presented in Table 3 provide the necessary information to calculate alternative comparisons that may be of interest.

5 The primary goal of the LPA in Study 2 was to determine whether the preferred, 5-profile solution selected in the previous study would produce similar profiles in an independent sample. However, an evaluation of competing 4- (AIC = 30,629.1; BIC = 30,789.4; BLRT *p* < 0.001; entropy = 0.94) and 6-profile models (AIC = 30,412.4; BIC = 30,640.2; BLRT *p* < 0.001; entropy = 0.95) continued to offer support for the preferred, replicated solution.

6 The 5-profile solution was again preferred to competing 4- (AIC = 22,377.8; BIC = 22,525.9; BLRT *p* < 0.001; entropy = 0.90) and 6-profile models (AIC = 22,125.7; BIC = 22,336.2; BLRT *p* < 0.001; entropy = 0.90).
