# Supplementary material for: Public beliefs about trauma and its consequences: Profiles and correlates of stigma
Source: Front Psychol. 2023 Jan 4;13:992574. doi: 10.3389/fpsyg.2022.992574 (PMC9846146; doi:10.3389/fpsyg.2022.992574)

Appendix A

Trauma Beliefs Scale

People may be exposed to stressful events during their lifetime. Some of these events can be classified as serious trauma (e.g., physical or sexual abuse, rape, exposure to death or injury during military service, involvement in accidents where someone was killed and/or seriously injured). Please read the following statements about the consequences of serious trauma. Rate each item as true or false based on your own beliefs or experiences. All responses are confidential, and there are no right or wrong answers. We are interested only in your personal opinion.

1. People exposed to serious trauma are generally unreliable.
2. **People exposed to serious trauma have healthy family relationships.**
3. People exposed to serious trauma often become dangerous.
4. **People exposed to serious trauma are as psychologically stable as they were before.**
5. People who have difficulty moving past serious trauma are generally faking.
6. People who have difficulty moving past serious trauma are generally emotionally weak.
7. **People exposed to serious trauma are generally fine.**
8. People exposed to serious trauma are unable to handle interpersonal relationships as effectively as before.
9. People exposed to serious trauma are unable to handle romantic relationships as effectively as before.
10. **People exposed to serious trauma are as trustworthy as they were before.**
11. People who have difficulty moving past serious trauma are generally making excuses for their own behavior.
12. **People exposed to serious trauma have healthy friendships.**
13. People exposed to serious trauma will never be the same.
14. People who have difficulty moving past serious trauma are generally looking for attention.
15. **People exposed to serious trauma have healthy romantic relationships.**
16. **People exposed to serious trauma rarely experience emotional difficulties.**
17. People who have difficulty moving past serious trauma are generally exaggerating their problems.
18. People exposed to serious trauma need to be on medication.
19. People exposed to serious trauma generally act strangely.
20. People exposed to serious trauma are changed forever.
21. **People exposed to serious trauma are good parents.**
22. People who have difficulty moving past serious trauma are unlikely to recover without professional help.
23. It is difficult to have a romantic relationship with someone who has experienced serious trauma.
24. People exposed to serious trauma often become violent.
25. It is difficult to work with someone who has experienced serious trauma.
26. I would be hesitant to interact with someone who has experienced serious trauma.
27. People exposed to serious trauma often become unpredictable.
28. People exposed to serious trauma are damaged.
29. People exposed to serious trauma are ruined.
30. People who have difficulty moving past serious trauma are generally trying to get something.

31. It is difficult for someone who has experienced serious trauma to be a good parent.
32. **It is difficult to identify someone who has experienced serious trauma.**
33. People exposed to serious trauma often become abusive.
34. I would be hesitant to develop a romantic relationship with someone who has experienced serious trauma.
35. People who have difficulty moving past serious trauma are generally lazy.
36. People exposed to serious trauma are broken.
37. People exposed to serious trauma need to be in therapy.
38. **People exposed to serious trauma are as reliable as they were before.**
39. People who have difficulty moving past serious trauma are generally crazy.
40. People exposed to serious trauma are unable to do their jobs as effectively as before.
41. **People exposed to serious trauma are the same people they were before.**
42. **People exposed to serious trauma generally recover on their own.**
43. People exposed to serious trauma are generally needy.
44. It is difficult to be friends with someone who has experienced serious trauma.
45. **People exposed to serious trauma generally do not need therapy.**
46. I would be hesitant to develop a friendship with someone who has experienced serious trauma.
47. People who have difficulty moving past serious trauma generally need support.
48. People exposed to serious trauma are generally untrustworthy.
49. **People exposed to serious trauma work effectively.**
50. People who have difficulty moving past serious trauma generally need treatment.
51. I would have difficulty connecting with someone who has experienced serious trauma.

Note: Bolded text identifies reverse coded items; items should be unbolded prior to administration

Domain Codes

Course:
- *C1-C7 are 13, 20, 28, 29, 36, 41r, 42r*

Dangerousness:
- *D1-D3 are 3, 24, 33*

Employability:
- *E1-E3 are 25, 40, 49r*

Mental Hygiene:
- *H1-H7 are 7r, 16r, 18, 22, 37, 45r, 50*

Moralizing:
- *M1-M9 are 5, 6, 11, 14, 17, 30, 35, 39, 43*

Social Concerns:
- *S1-S15 are 2r, 8, 9, 12r, 15r, 21r, 23, 26, 31, 32r, 34, 44, 46, 47, 51*

Predictability:
- *P1-P7 are 1, 4r, 10r, 19, 27, 38r, 48*

Supplemental Table A

Item-Level Endorsement for Studies 1 to 3.

Code	Num	Item	S1	S2	S3
**c1**	*13*	People exposed to serious trauma will never be the same.	61.4	64.3	71.4
**c2**	*20*	People exposed to serious trauma are changed forever.	74.0	74.3	83.0
**c3**	*28*	People exposed to serious trauma are damaged.	47.0	40.2	41.5
**c4**	*29*	People exposed to serious trauma are ruined.	3.2	3.4	5.0
**c5**	*37*	People exposed to serious trauma are broken.	26.7	15.9	20.6
**c6**	*42r*	***People exposed to serious trauma are the same people they were before.***	66.6	70.5	65.1
**c7**	*43r*	***People exposed to serious trauma generally recover on their own.***	73.0	76.9	69.0
		**Mean**	***50.4***	***49.8***	***51.0***
**d1**	*3*	People exposed to serious trauma often become dangerous.	24.3	22.5	23.6
**d2**	*24*	People exposed to serious trauma often become violent.	24.0	20.7	22.8
**d3**	*33*	People exposed to serious trauma often become abusive.	14.6	12.0	21.2
		**Mean**	***21.0***	***19.0***	***22.8***
**e1**	*25*	It is difficult to work with someone who has experienced serious trauma.	24.0	23.9	26.4
**e2**	*41*	People exposed to serious trauma are unable to do their jobs as effectively as before.	23.3	19.8	27.5
**e3**	*50r*	***People exposed to serious trauma work effectively.***	33.4	30.7	23.6
		**Mean**	***27.0***	***25.3***	***25.9***
**h1**	*7r*	***People exposed to serious trauma are generally fine.***	82.3	84.3	73.6
**h2**	*16r*	***People exposed to serious trauma rarely experience emotional difficulties.***	88.6	91.2	90.1
**h3**	*18*	People exposed to serious trauma need to be on medication.	14.6	11.6	25.8
**h4**	*22*	People who have difficulty moving past serious trauma are unlikely to recover without professional help.	45.5	46.2	68.1
**h5**	*38*	People exposed to serious trauma need to be in therapy.	48.3	39.6	68.1
**h6**	*46r*	***People exposed to serious trauma generally do not need therapy.***	84.2	82.9	82.1
**h7**	*51*	People who have difficulty moving past serious trauma generally need treatment.	74.5	76.9	88.7
		**Mean**	***62.8***	***62.8***	***71.4***
**m1**	*5*	People who have difficulty moving past serious trauma are generally faking.	4.5	4.8	4.7
**m2**	*6*	People who have difficulty moving past serious trauma are generally emotionally weak.	24.3	20.1	16.8
**m3**	*11*	People who have difficulty moving past serious trauma are generally making excuses for their own behavior.	12.6	11.2	7.7
**m4**	*14*	People who have difficulty moving past serious trauma are generally looking for attention.	5.0	3.8	5.8
**m5**	*17*	People who have difficulty moving past serious trauma are generally exaggerating their problems.	6.9	4.4	5.2

**m6**	*30*	People who have difficulty moving past serious trauma are generally trying to get something.	7.4	4.8	4.7
**m7**	*36*	People who have difficulty moving past serious trauma are generally lazy.	4.2	3.2	4.1
**m8**	*40*	People who have difficulty moving past serious trauma are generally crazy.	4.7	3.2	7.1
**m9**	*44*	People exposed to serious trauma are generally needy.	20.1	13.2	19.2
		**Mean**	***10.0***	***7.7***	***8.5***
**s1**	*2r*	***People exposed to serious trauma have healthy family relationships.***	44.3	44.4	38.5
**s2**	*8*	People exposed to serious trauma are unable to handle interpersonal relationships as effectively as before.	54.2	52.6	65.1
**s3**	*9*	People exposed to serious trauma are unable to handle romantic relationships as effectively as before.	50.3	53.4	61.3
**s4**	*12r*	***People exposed to serious trauma have healthy friendships.***	28.5	28.9	21.2
**s5**	*15r*	***People exposed to serious trauma have healthy romantic relationships.***	43.6	35.7	35.7
**s6**	*21r*	***People exposed to serious trauma are good parents.***	24.5	18.1	17.6
**s7**	*23*	It is difficult to have a romantic relationship with someone who has experienced serious trauma.	45.1	47.8	56.3
**s8**	*26*	I would be hesitant to interact with someone who has experienced serious trauma.	15.4	11.2	14.0
**s9**	*31*	It is difficult for someone who has experienced serious trauma to be a good parent.	15.1	15.1	20.1
**s10**	*32r*	***It is difficult to identify someone who has experienced serious trauma.***	24.8	20.1	28.9
**s11**	*34*	I would be hesitant to develop a romantic relationship with someone who has experienced serious trauma.	27.7	23.1	32.1
**s12**	*45*	It is difficult to be friends with someone who has experienced serious trauma.	17.6	16.7	21.4
**s13**	*47*	I would be hesitant to develop a friendship with someone who has experienced serious trauma.	9.7	8.0	12.9
**s14**	*48*	People who have difficulty moving past serious trauma generally need support.	90.4	90.4	90.3
**s15**	*52*	I would have difficulty connecting with someone who has experienced serious trauma.	17.8	12.6	18.1
		**Mean**	***34.1***	***32.6***	***35.9***
**p1**	*1*	People exposed to serious trauma are generally unreliable.	9.2	7.8	9.1
**p2**	*4r*	***People exposed to serious trauma are as psychologically stable as they were before.***	90.1	90.8	84.9
**p3**	*10r*	***People exposed to serious trauma are as trustworthy as they were before.***	29.2	29.3	20.9
**p4**	*19*	People exposed to serious trauma generally act strangely.	36.4	26.7	27.5
**p5**	*27*	People exposed to serious trauma often become unpredictable.	36.4	31.1	38.5
**p6**	*39r*	***People exposed to serious trauma are as reliable as they were before.***	37.1	37.3	32.7
**p7**	*49*	People exposed to serious trauma are generally untrustworthy.	5.9	4.4	7.1
		**Mean**	***35.0***	***32.8***	***31.9***

Note: Items in bold font reverse-coded for analyses

Supplemental Figure A

Domain Means, Profile Scores, and 95% Confidence Bounds for Study 1.

1. ***Sympathizing***
2. ***Performance-Focused***
3. ***Safety-Focused***
4. ***Fearful***
5. ***Pejorative***

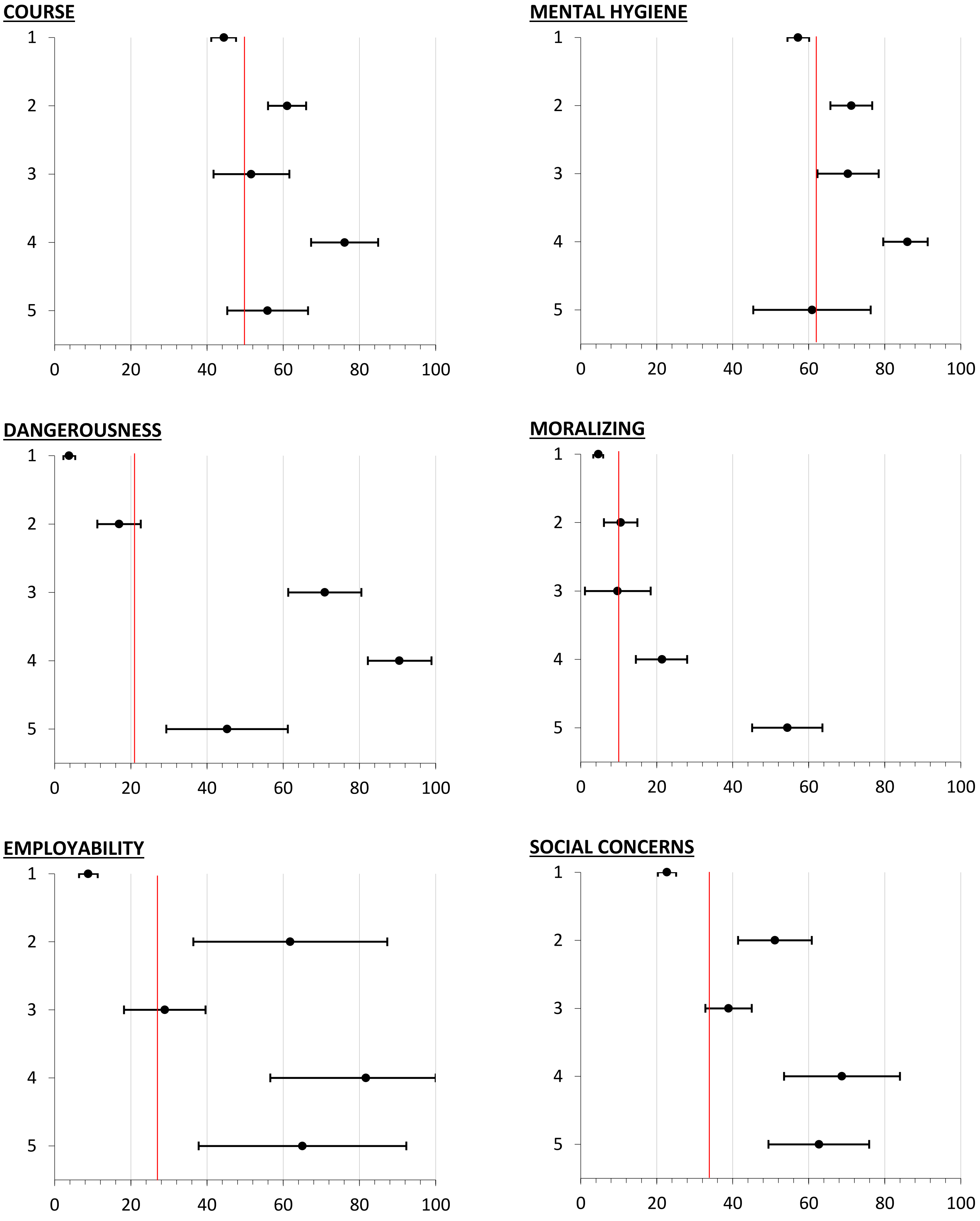

PREDICTABILITY

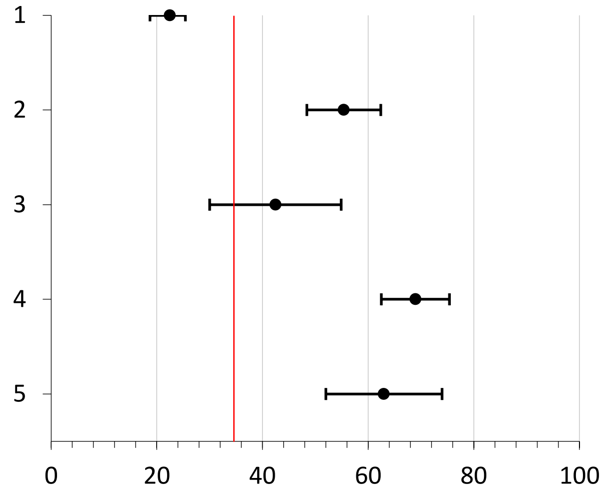

Note: Vertical red line indicates overall sample mean (N = 404)

Supplemental Figure B

Domain Means, Profile Scores, and 95% Confidence Bounds for Study 2.

1. ***Sympathizing***
2. ***Performance-Focused***
3. ***Safety-Focused***
4. ***Fearful***
5. ***Pejorative***

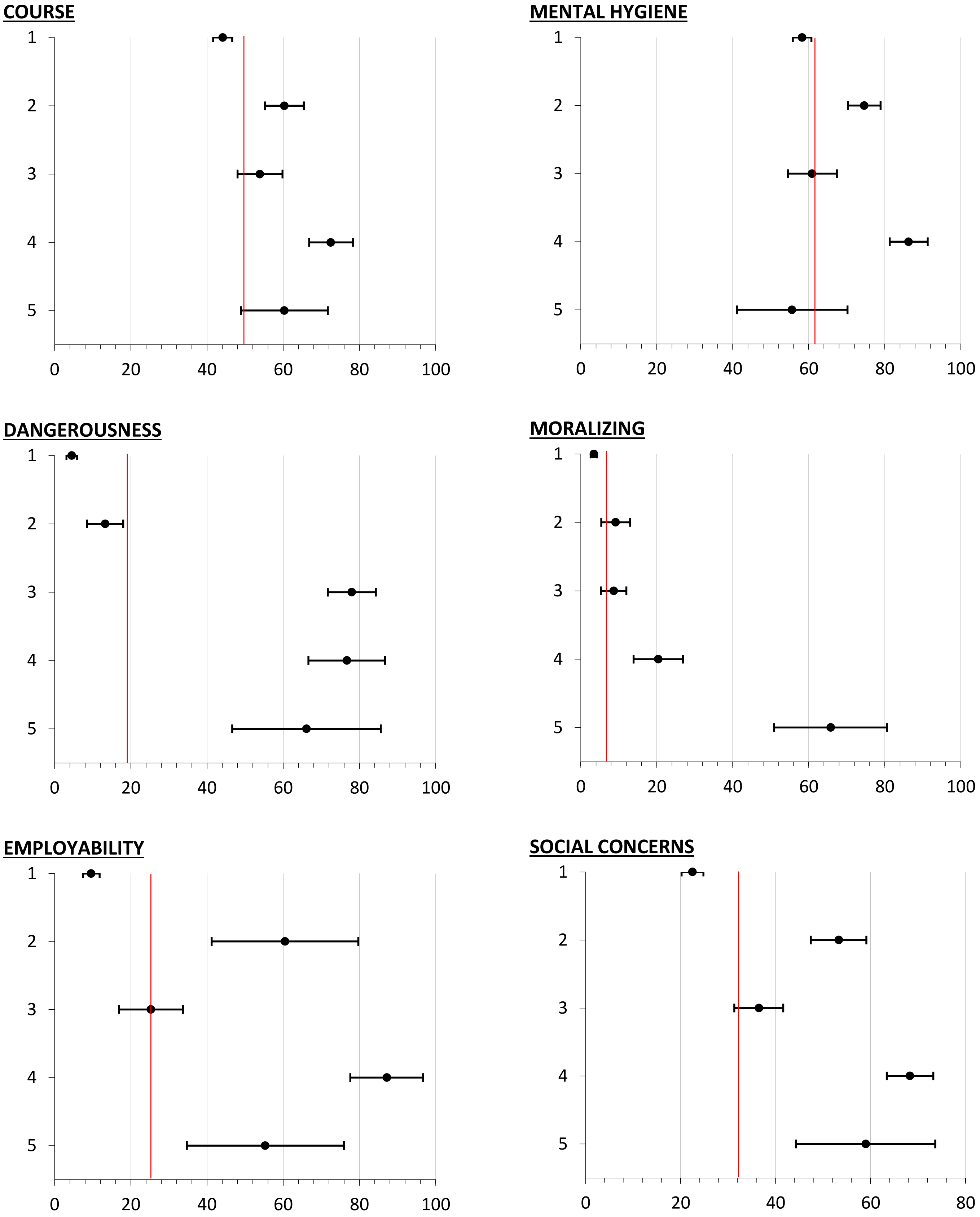

PREDICTABILITY

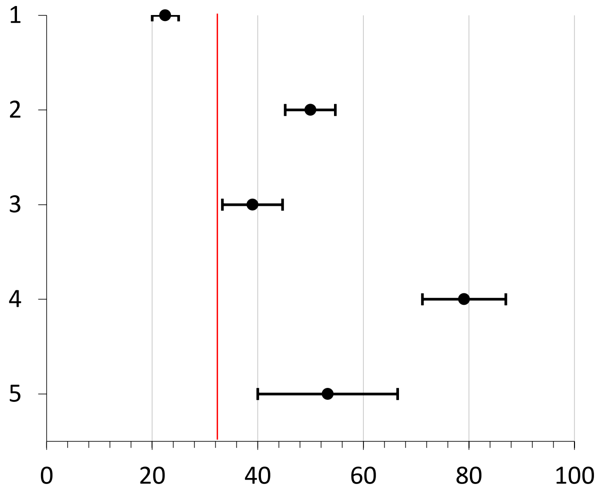

Note: Vertical red line indicates overall sample mean (*N* = 502)

1. ***Sympathizing***
2. ***Performance-Focused***
3. ***Safety-Focused***
4. ***Fearful***
5. ***Pejorative***

Supplemental Figure C

Domain Means, Profile Scores, and 95% Confidence Bounds for Study 3.

<u>COURSE</u>

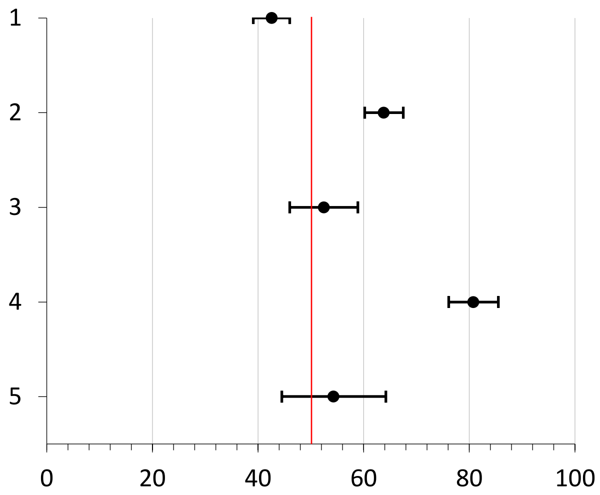

<u>MENTAL HYGIENE</u>

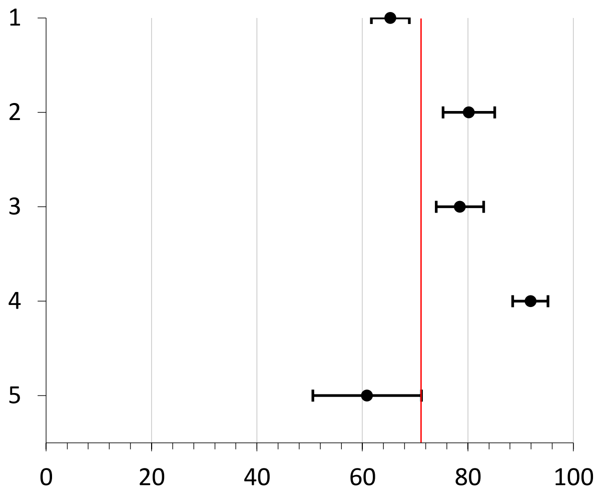

<u>DANGEROUSNESS</u>

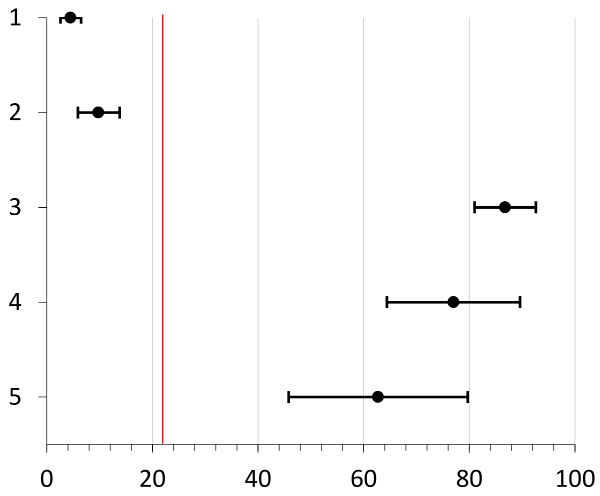

<u>MORALIZING</u>

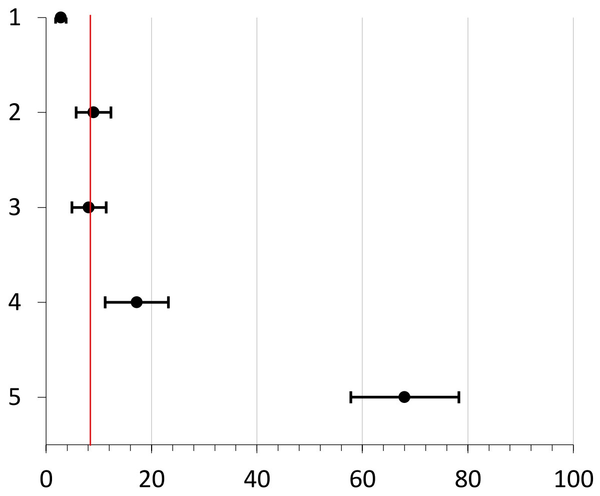

<u>EMPLOYABILITY</u>

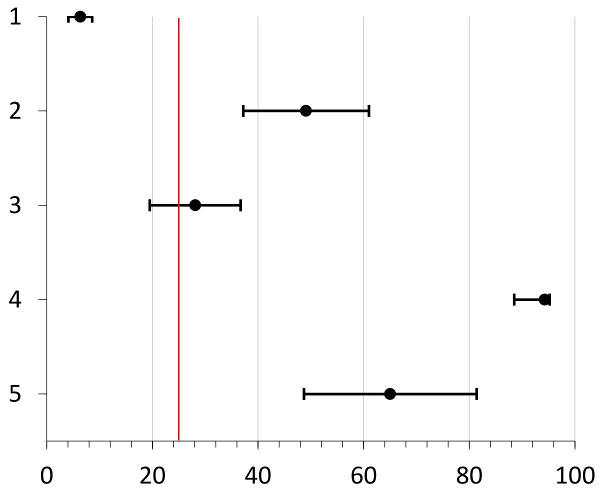

<u>SOCIAL CONCERNS</u>

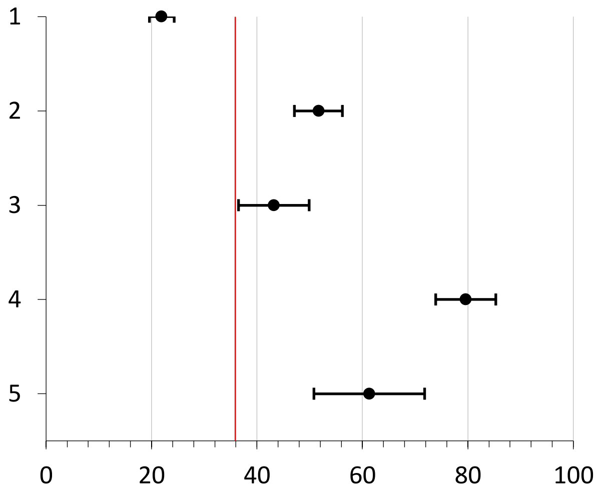

PREDICTABILITY

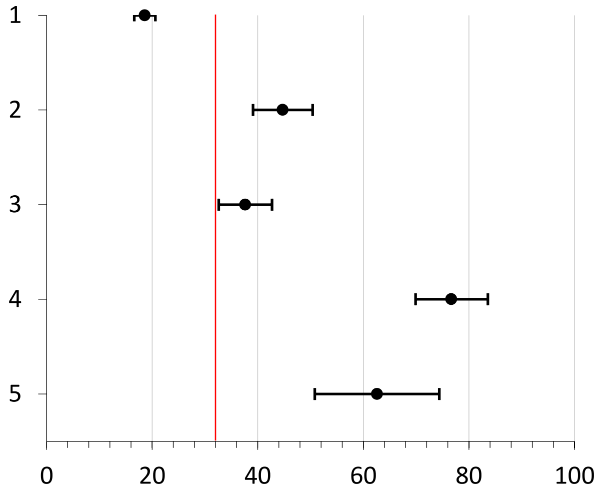

Note: Vertical red line indicates overall sample mean (*N* = 364)

Supplemental Figure D

CAMI Scores and 95% Confidence Bounds for Study 2 and Study 3.

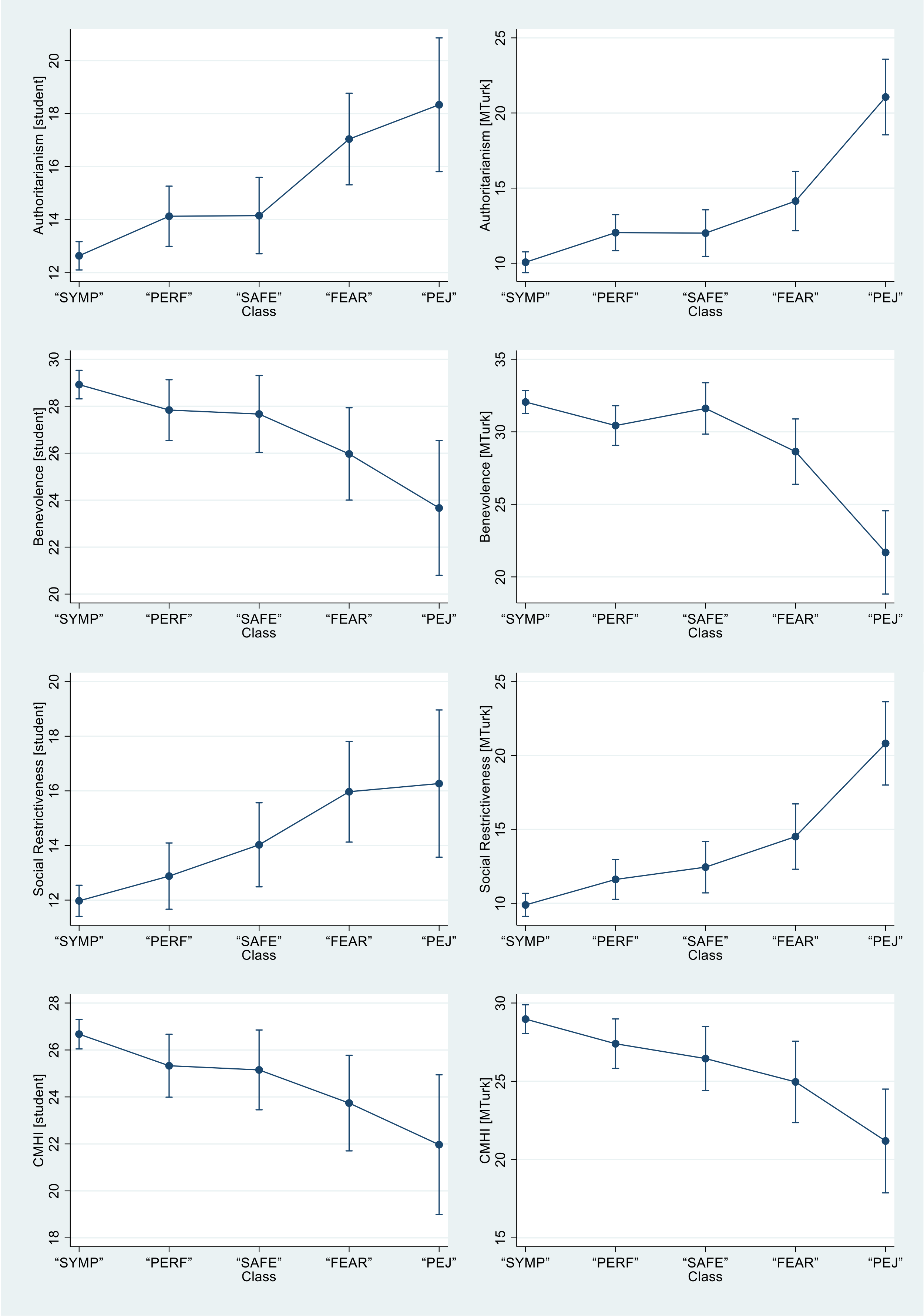

Supplement: Supplementary file 1 [file Data_Sheet_1.pdf]
